# Intergenerational transmission of adverse childhood experiences: roles of parental depressive symptoms and parenting stress

**DOI:** 10.1186/s13034-025-01001-4

**Published:** 2025-12-24

**Authors:** Mingxiao Liu, Aiyi Liu, Ling Guo, Jiefeng Ying, Xinchun Wu

**Affiliations:** 1 Beijing Key Laboratory of Applied Experimental Psychology, National Demonstration Center for Experimental Psychology Education (Beijing Normal University), Faculty of Psychology, Beijing Normal University, No. 19 Xinjiekouwai Street, Beijing, 100875 China; 2 CAS Key Laboratory of Mental Health, Institute of Psychology, Chinese Academy of Sciences, Beijing, 100101 China; 3 Department of Psychology, University of the Chinese Academy of Sciences, Beijing, 100049 China; 4 School of Applied Psychology , Beijing Normal University at Zhuhai , Zhuhai, 519087 Guangdong China

**Keywords:** Intergenerational transmission, Adverse childhood experiences, Parental depressive symptoms, Parenting stress, Adolescents

## Abstract

**Background:**

Parents’ adverse childhood experiences (ACEs) are associated with poorer mental health and elevated parenting stress, which could increase their children’s risk of ACE exposure. Accordingly, understanding the mechanisms underlying the intergenerational transmission of ACEs is crucial for breaking this cycle.

**Objective:**

Based on family systems theory and parenting stress model, this study aims to explore the sequential mediating role of parental depressive symptoms and parenting stress in the association between parental ACEs and adolescents’ ACEs.

**Methods:**

This cross-sectional study investigated 1479 Chinese families. Fathers and mothers completed self-report questionnaires assessing ACEs, depressive symptoms, and parenting stress independently, while their children (adolescents) filled out the ACEs assessment questionnaire only.

**Results:**

This study found that both paternal and maternal ACEs had total effects on adolescents’ ACEs. In addition, maternal ACEs were indirectly associated with adolescents’ ACEs via maternal parenting stress. Moreover, both paternal and maternal ACEs were associated with maternal, but not paternal, depressive symptoms and parenting stress, which in turn contributed to adolescents’ ACEs.

**Conclusions:**

Targeting maternal depressive symptoms and parenting stress may offer a feasible entry point to interrupt intergenerational ACE transmission. These findings support school-based integrated screening and referral for students and caregivers, with attention to maternal depression and parenting stress.

## Introduction

Approximately 60% of adults have at least one adverse childhood experience (ACE) [29]. The enduring repercussions of ACEs are evident in adulthood as adverse mental and physical health outcomes and behavioral problems [47]. Moreover, these repercussions may be transmitted intergenerationally. Parents with ACEs often replicate child-rearing approaches reminiscent of their own upbringing [54]. Accordingly, individuals exposed to ACEs are more likely to have children who experience similar adversities [61, 65]. This transmission might stem from parental psychopathological symptoms (e.g., depressive symptoms), which places greater demands on coping resources and increases parenting stress. Although a growing literature has examined intergenerational pathways of ACEs (e.g., Langevin et al., [39], evidence that integrates the Chinese cultural context while explicitly including fathers and leveraging a large adolescent sample remains scarce. Recognizing that fathers and mothers might influence their children in different ways [27, 36], this research examines their roles in their children’s upbringing separately, given the importance of understanding the transmission mechanisms for developing and implementing targeted prevention measures.

### Intergenerational transmission of aces

ACEs encompass a range of detrimental events and trauma occurring before the age of 18, ranging from abuse, neglect, and household dysfunctions [24]. Multiple empirical studies have found a positive link between parents’ and children’s ACE scores [61, 65]. In a meta-analysis of 84 studies reporting on 285 effect sizes found that children of parents who experienced abuse and neglect in their own childhoods were three times more susceptible to enduring similar abuse or neglect, compared to those whose parents did not have these experiences [6]. In addition, children whose parents reported four or more ACEs were subject to a three-fold higher risk of undergoing at least four ACEs themselves [61].

Existing research on the intergenerational transmission of ACEs has predominantly focused on mothers who have traditionally been viewed as primary caregivers [53, 65]. In some cases, studies have also aggregated maternal and paternal data, thereby obscuring potential differences between the two parental roles [73]. However, an increasing body of studies have rightfully acknowledged the unique role that fathers play in children’s development [7, 13]. Furthermore, paternal and maternal ACEs have different effects on adolescents’ ACEs. A study found that maternal ACEs were more strongly associated with their children’s ACEs than paternal ACEs [61]. In contrast, another study found that the correlation between paternal ACEs and their adult children’s ACEs is statistically significant, but not the association among mother-child dyads [64].

In the context of Chinese culture, parenting practices have long been shaped by Confucian ideologies that emphasize family hierarchy, filial piety, and the maintenance of harmony through interdependence and role fulfillment [41, 59]. Traditionally, there has been a clear gendered division of labor: fathers have typically occupied the role of economic providers and authority figures, while mothers have been primarily responsible for caregiving and emotional support [22]. However, with the rapid social and economic transformation in contemporary China, the family structure has been undergoing notable changes, including increasing maternal participation in the workforce and greater paternal involvement in childcare [43]. These changes underscore the importance of examining maternal and paternal pathways separately. Therefore, it is important to incorporate both parental and maternal ACEs into the same model for a comprehensive understanding of their combined impact on adolescents’ ACEs.

### Sequential indirect paths of parental depressive symptoms and parenting stress

According to the parenting stress model, the origins of stress are multifaceted and encompass various dimensions, including parent psychopathology [1, 19]. Within this framework, parental symptomology, particularly depressive symptoms, is identified as a more significant predictor in causing parenting stress than either PTSD symptoms or trauma exposure among parents [45]. Empirical studies found that parental depressive symptoms predicted parenting distress [4, 18, 60]. Therefore, this study aims to explore the sequential indirect pathways of parental depressive symptoms and parenting stress in the intergenerational transmission of ACEs.

### Role of parental depressive symptoms

Family systems theory highlights the interplay of behaviors and emotions among family members, considering the family as a tightly interconnected system underpinned by a collection of subsystems such as the parental subsystem, where one subsystem can have ramifications for the functioning of the whole system [15]. Before children are born, their parents have already been influenced by the parents of their parents (i.e., grandparents from the children’s perspective). These early experiences influence how parents (i.e., members of the caregiver subsystem) relate to and raise their own children. Within this framework, ACEs have been found to increase vulnerability to depressive symptoms in adulthood, which can impair emotional responsiveness, reduce sensitivity, and compromise parents’ ability to provide consistent care. Such impairments may, in turn, heighten the risk of neglectful or harsh parenting, contributing to the occurrence of ACEs in the next generation. Empirical studies have shown, for instance, that maternal depression may function as a key mechanism linking parental ACEs to intergenerational maltreatment [14, 71].

A growing body of research emphasizes the lasting impact of ACEs on depression throughout adulthood [21, 46]. A meta-analysis revealed that the risk odds indicating the risk association between ACEs and depressive symptoms rise as the number of ACEs increases, possibly demonstrating a dose-response relationship of ACEs [50]. Additionally, research has demonstrated the intergenerational effect of parental ACEs through parental psychopathological pathways (e.g., depressive symptoms) on children’ ACEs [8, 48, 66]. In this context, it is reasonable to infer that depressive symptoms could serve as a mechanism through which the intergenerational transmission of ACEs occurs.

### Role of parenting stress

Drawing from family systems theory [15], parenting behavior also assumes a significant role in the caregiver’s subsystem. Parenting stress is the mental strain that arises when parents perceive a mismatch between parenting demands and their available resources [19]. Previous studies have consistently shown a link between ACEs and parenting stress, highlighting this relationship across various investigations [49, 55, 72]. Notably, a dose–response relationship has been identified concerning ACEs and parenting stress [38, 55]. Indeed, when individuals with ACEs within the family context transition into the role of parents, the challenges of nurturing their own children may become formidable, leading to heightened parenting stress [49, 72].

While extensive research has uncovered the correlation between parental ACEs and parenting stress, there is still a relative scarcity of studies examining the link between parenting stress and children’s exposure to ACEs. Nonetheless, there is still noteworthy research evidence supporting this connection. Parents experiencing exceptionally high and/or prolonged levels of parenting stress often exhibit heightened demands, adopt less responsive parenting styles, and engage in diminished communication with their children [16, 52]. These behaviors are correlated with child neglect or abuse, aligning with the dimensions encompassed in the ACE definition. One empirical study has identified that within households, children with parents indicating “high parenting stress” have a higher probability of encountering four or more ACEs than those from homes with “low parenting stress” [17]. Given these findings, it appears plausible to suggest that parenting stress may serve as a mechanism in the transmission of ACEs from one generation to the next.

### Actor-partner interdependence model for intergenerational ACE pathways: actor and partner effects of parental depressive symptoms and parenting stress

In addition, family members influence each other per the family systems theory. One parent’s ACEs may cross over into the other parent’s depressive symptoms and parenting stress in a family system. The actor-partner interdependence model (APIM) sheds light on the reciprocal interactions between fathers and mothers [35]. Prior research has demonstrated the relevance of APIM in family-based studies of psychological distress, parenting, and couple dynamics [2, 57], yet little is known about how such dyadic pathways operate in the context of intergenerational ACE transmission. This model facilitates a concurrent analysis of how one parent’s ACEs affect both their own depressive symptoms and parenting stress (the actor effect) and on the partner’s depressive symptoms and parenting stress (the partner effect). It further tests whether these pathways are associated with adolescents’ ACEs.

### Current study

Based on family systems theory and parenting stress model, this study proposes the following hypotheses: (1) Both paternal ACEs and maternal ACEs would positively predict adolescents’ ACEs; (2) Parental depressive symptoms and parenting stress would serve as partial mediators between parental ACEs and adolescents’ ACEs; (3) Parental depressive symptoms and parental parenting stress would function as a sequential mediating chain in the relationship between parental ACEs and adolescents’ ACEs; (4) Parental (paternal and maternal) depressive symptoms and parenting stress would indirectly link both actor and partner ACEs to adolescents’ ACEs.

## Methods

### Participants

This cross-sectional study involved 1503 families, including fathers, mothers, and their adolescent offspring. Due to the use of paper-based questionnaires for adolescent assessments, some demographic variables were missing. As a result, 1479 families were included in the final structural equation modeling analysis, as cases with missing demographic data were excluded via listwise deletion. The mean age of the 1479 adolescents investigated was 13.37 years old (*SD* = 1.59), with 57.5% being girls. Furthermore, the parents’ educational levels were as follows: junior middle school or below; fathers: 397 (26.8%); mothers: 447 (30.2%); high school or technical secondary school, fathers: 485 (32.8%), mothers: 448 (30.3%); and college or above, fathers: 597 (40.4%) mothers: 584 (39.5%). Fathers had an average age of 42.93 years (*SD* = 5.00), while mothers averaged 41.45 years (*SD* = 4.74).

### Procedures

Before recruiting participants, the research was approved by research ethics committee of the institution of the first author. This study was conducted from June to November 2023 in 11 schools across Henan, Guangdong, and the Inner Mongolia, China. Adolescent students and their parents were invited to participate in the study. Prior to initiating data collection, informed consent was obtained from teachers, students, and parents. Parents completed the questionnaires using an online platform called “Questionnaire Star”, while the adolescents filled out the paper-based survey manually in classrooms during class hours, with each class serving as a unit and guidance provided by their psychological teachers. School psychological teachers, who had received study-specific training, provided standardized instructions and supervision. Participants could ask the teachers questions in class or online if anything was unclear. Participants were anonymous and their information was confidential. Every participant was allowed to withdraw from the study at any time without any negative consequences. School psychologists were also available to provide follow-up counseling services to participants upon request.

### Measures

#### ACEs

This study used the ACE questionnaire adapted for Chinese populations to identify both parental and their children’s adversity [23, 24, 42]. The ACE questionnaire consists of 29 items, including: emotional abuse; physical abuse; sexual abuse; emotional neglect; physical neglect. Family dysfunction includes domestic violence, presence of substance abusers in the home, presence of individuals with mental health issues in the home, parental separation or divorce, and presence of a criminal family member. Following prior work [23], each of the 10 ACE categories was coded 0/1 (absent/present) and summed to yield a total score (range = 0–10). Fathers, mothers, and adolescents rated the ACE items to indicate the extent to which each item was true for them before age 18. In this study, the values of Cronbach’s alpha of the scale were 0.86 for fathers, 0.87 for mothers, and 0.85 for adolescents.

#### Depressive symptoms

To measure depressive symptoms during the past two weeks, this study used the Patient Health Questionnaire-9 (PHQ-9; Kroenke et al., [37]. The Chinese version of the PHQ-9, which has been validated in previous research with Chinese populations (e.g., Wang et al., [70], was used in this study. Parents were asked to assess the scale comprising 9 items (e.g., “Feeling down, depressed, or hopeless”) on a 4-point scale ranging from 0 (*not at all*) to 3 (*nearly every day*). A higher total score indicates a higher level of depressive symptoms, with scores ranging from 0 to 27. In this study, the values of Cronbach’s alpha of the scale were 0.93 for mothers and 0.92 for fathers.

#### Parenting stress

To measure parenting stress, the Chinese revised version of the Parenting Stress Index–Short Form (PSI-SF) was used [1]; Luo et al., 2021). The PSI-SF consists of three subscales: parental distress (5 items, e.g., “I no longer have interest in some things like I used to.”), parent-child dysfunctional interaction (5 items, e.g., “Most of the time, I feel like my child doesn’t like me.”), and difficult child (5 items, e.g., “Sometimes the things my child does make me angry.”). Each item was rated on a 5-point scale, ranging from 1 (*strongly disagree*) to 5 (*strongly agree*). A higher total score indicates a higher level of parenting stress, with scores ranging from 15 to 75. In this study, the values of Cronbach’s alpha of the scale were 0.94 for mothers and 0.94 for fathers. The confirmatory factor analysis indicated acceptable construct validity for paternal parenting stress (*χ*^2^(87) = 1181.186, RMSEA = 0.092, CFI = 0.928, TLI = 0.913, SRMR = 0.041), with all standardized item loadings significant (*p* < 0.001) and ranging from 0.703 to 0.851. Similarly, the model showed acceptable fit for mothers (*χ*^2^(87) = 1268.625, RMSEA = 0.096, CFI = 0.918, TLI = 0.902, SRMR = 0.044), with significant standardized loadings ranging from 0.678 to 0.856.

### Statistical analysis

The statistical analysis in this study involved three steps. First, parents were required to answer all items on the scheduled online survey, no data were lost. Adolescents filled out the paper-based questionnaire, and the missing rate for each item was below 5%, and missing data, except demographic variables (e.g., sex), were handled using Mean Imputation in SPSS 26.0. Subsequently, the total scores for each variable were calculated, and descriptive statistics along with Spearman correlation analysis were conducted. Second, using structural equation modeling (SEM) in Mplus 8.3, we tested a sequential path model in which paternal and maternal depressive symptoms and parenting stress served as indirect pathways linking each parent’s ACEs to adolescents’ ACEs. In the SEM, parenting stress was treated as latent variables based on subscale scores, while ACEs and depressive symptoms were treated as observable variables using total scores. Furthermore, we adopted the Actor-Partner Interdependence Model (APIM) framework to examine both actor and partner effects within the family system. Specifically, we tested whether each parent’s ACEs predicted their own depressive symptoms and parenting stress (actor effects), as well as their partner’s depressive symptoms and parenting stress (partner effects), and adolescents’ ACEs. Differences between maternal and paternal path coefficients were tested using the MODEL CONSTRAINT function in Mplus. Third, we conducted the Bootstrapping analysis to test the mediating effects in our proposed model, which entailed the generation of 5000 bootstrapping samples through random sampling from the original dataset. In addition, all the direct and mediating analysis controlled for adolescents’ sex, age and both fathers’ and mothers’ educational levels.

## Results

### Descriptive statistics

Table 1 presents the variables’ means, standard deviations, and Spearman correlations. As expected, all the variables were significantly correlated, which provided the initial support for the hypothesized sequential path model.

**Table 1:** Means, standard deviations, and spearman correlations among variables. The numbers indicated standardized regression coefficients. ^*^p < 0.05, ^***^p < 0.001.

Variable	1	2	3	4	5	6	7
1. Adolescents’ ACEs	1						
2. Paternal ACEs	0.140^***^	1					
3. Maternal ACEs	0.155^***^	0.242^***^	1				
4. Paternal depressive symptoms	0.102^***^	0.240^***^	0.098^***^	1			
5. Maternal depressive symptoms	0.112^***^	0.138^***^	0.226^***^	0.303^***^	1		
6. Paternal parenting stress	0.097^***^	0.253^***^	0.113^***^	0.369^***^	0.149^***^	1	
7. Maternal parenting stress	0.101^***^	0.116^***^	0.236^***^	0.160^***^	0.394^***^	0.375^***^	1
Mean	0.927	0.737	0.659	2.174	2.497	31.028	31.882
SD	1.458	1.175	1.200	3.545	3.932	11.241	10.929

*N* = 1479. ^***^p < 0.001

### Sequential path model

**Fig. 1:**
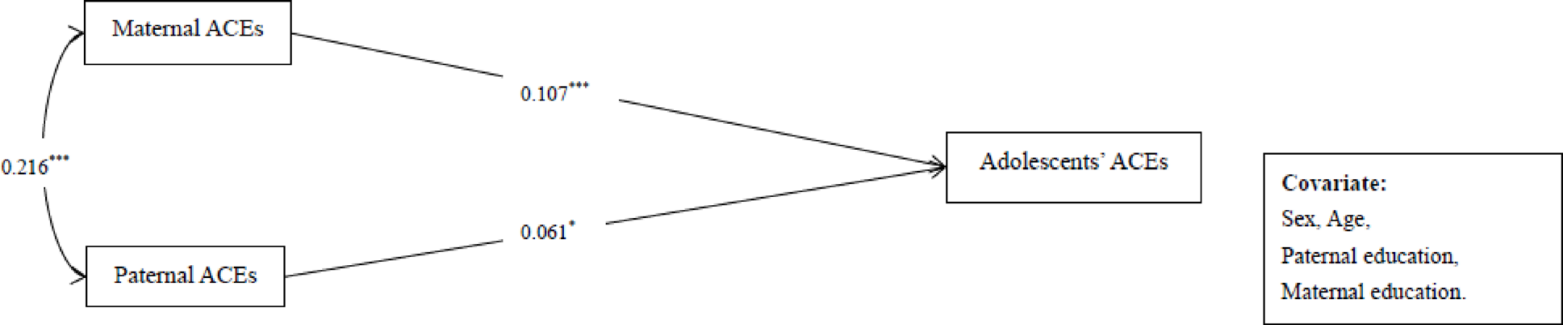
Standardized path coefficients of the total effect

**Fig. 2:**
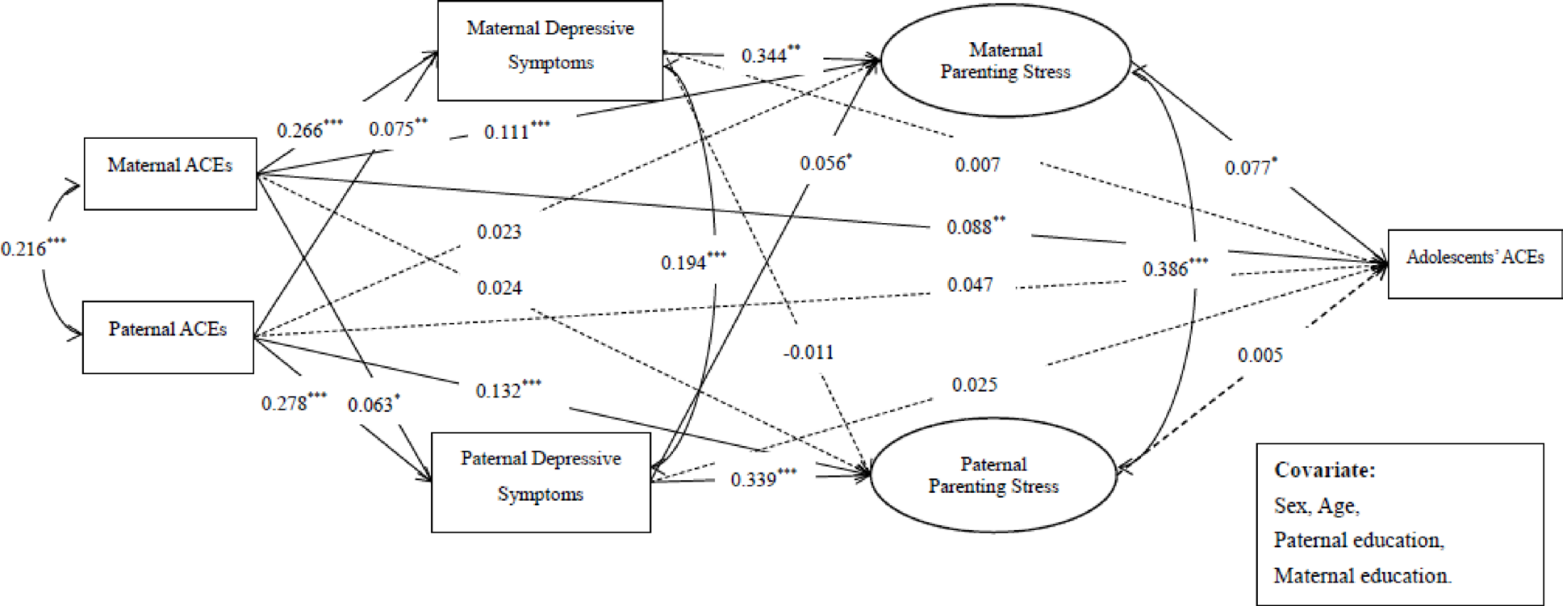
Standardized path coefficients of the sequential path model

Significant and positive total effects on adolescents’ ACEs (Fig. 1) were found for both paternal (β = 0.061; *p* = 0.029) and maternal ACEs (β = 0.107; *p* < 0.001), although the difference between these effects was not significant (β_*diff*_ = 0.055, *p* = 0.319). We further examined the mechanism of paternal and maternal depressive symptoms, as well as parenting stress, in the intergenerational transmission of ACEs. The hypothesized sequential path model had acceptable fitting indices: *χ*^2^(52) = 329.338, CFI = 0.952, TLI = 0.916, RMSEA = 0.060, SRMR = 0.026. Figure 2 reports the standardized path coefficients of the hypothesized sequential path model. Specifically, maternal ACEs were not only positively related to maternal depressive symptoms (β = 0.266, *p* < 0.001), maternal parenting stress (β = 0.111, *p* < 0.001), and adolescent ACEs (β = 0.088, *p* = 0.004), but also to paternal depressive symptoms (β = 0.063, *p* = 0.039). Paternal ACEs were positively related to paternal (β = 0.278, *p* < 0.001) and maternal (β = 0.075, *p* = 0.008) depressive symptoms, and paternal parenting stress (β = 0.132, *p* < 0.001). Also, maternal depressive symptoms were positively related to their own parenting stress (β = 0.344, *p* < 0.001), and paternal depressive symptoms were positively related to their parenting stress (β = 0.339, *p* < 0.001). In addition, maternal parenting stress was positively related to adolescents’ ACEs (β = 0.077, *p* = 0.025).

To further examine differences in maternal and paternal transmission pathways, parameter constraints were specified within the SEM to compare corresponding path coefficients. The differences in the direct effects of maternal versus paternal ACEs (β_*diff*_ = 0.049, *p* = 0.398), depressive symptoms (β_*diff*_ =–0.007, *p* = 0.701), and parenting stress (β_*diff*_ = 0.036, *p* = 0.180) on adolescents’ ACEs were not statistically significant. Parameter comparisons further confirmed that actor effects were consistently stronger than partner effects across most pathways. Specifically, maternal ACEs had a significantly greater impact on maternal depressive symptoms (β_*diff*_ = 0.688, *p* < 0.001) and maternal parenting stress (β_*diff*_ = 0.206, *p* = 0.012) than on paternal outcomes. Similarly, paternal ACEs more strongly predicted paternal depressive symptoms (β_*diff*_ = 0.589, *p* < 0.001) and paternal parenting stress (β_*diff*_ = 0.303, *p* = 0.001) than maternal outcomes. Maternal depressive symptoms had a larger influence on maternal parenting stress than cross-partner influence (β_*diff*_ = 0.264, *p* < 0.001).

Table 2 presents the results of the Bootstrap tests of the mediating effects in the overall model. As shown, maternal parenting stress indirectly related maternal ACEs to adolescents’ ACEs, and maternal depressive symptoms with subsequent parenting stress likewise linked both paternal and maternal ACEs to adolescents’ ACEs.

**Table 2:** Bootstrapping analysis of the mediating effects

Indirect effect	Total sample	
	Estimate	95%CI
Total effect	**0.061**	**[0.008**,** 0.117]**
Direct effect	0.047	[−0.009, 0.104]
Paternal ACEs – Paternal depressive symptoms – Adolescents’ ACEs	0.007	[−0.009, 0.024]
Paternal ACEs – Maternal depressive symptoms – Adolescents’ ACEs	0.001	[−0.004, 0.007]
Paternal ACEs – Paternal parenting stress – Adolescents’ ACEs	0.001	[−0.008, 0.009]
Paternal ACEs – Maternal parenting stress – Adolescents’ ACEs	0.002	[−0.002, 0.008]
Paternal ACEs – Paternal depressive symptoms – Paternal Parenting Stress – Adolescents’ ACEs	0.000	[−0.005, 0.006]
Paternal ACEs – Maternal depressive symptoms – Paternal Parenting Stress – Adolescents’ ACEs	0.000	[0.000, 0.000]
Paternal ACEs – Paternal depressive symptoms – Maternal Parenting Stress – Adolescents’ ACEs	0.001	[0.000, 0.004]
Paternal ACEs – Maternal depressive symptoms –Maternal Parenting Stress – Adolescents’ ACEs	**0.002**	**[0.000**,** 0.005]**
Total effect	**0.107**	**[0.050**,** 0.169]**
Direct effect	**0.088**	**[0.028**,** 0.149]**
Maternal ACEs – Paternal depressive symptoms – Adolescents’ ACEs	0.002	[−0.001, 0.008]
Maternal ACEs – Maternal depressive symptoms – Adolescents’ ACEs	0.002	[−0.014, 0.019]
Maternal ACEs – Paternal parenting stress – Adolescents’ ACEs	0.000	[−0.002, 0.003]
Maternal ACEs – Maternal parenting stress – Adolescents’ ACEs	**0.009**	**[0.002**,** 0.021]**
Maternal ACEs – Paternal depressive symptoms – Paternal parenting stress – Adolescents’ ACEs	0.000	[−0.001, 0.002]
Maternal ACEs – Maternal depressive symptoms – Paternal parenting stress – Adolescents’ ACEs	0.000	[−0.001, 0.000]
Maternal ACEs – Paternal depressive symptoms – Maternal parenting stress – Adolescents’ ACEs	0.000	[0.000, 0.001]
Maternal ACEs – Maternal depressive symptoms –Maternal parenting stress – Adolescents’ ACEs	**0.007**	**[0.001**,** 0.014]**

## Discussion

Based on family systems theory and parenting stress model (Bandura, 1973, 1977; Cox & Paley [15]), this study explored the direct and indirect links between parental ACEs and adolescents’ ACEs by delving into parental depressive symptoms and parenting stress. The main findings were as follows: (1) Both paternal and maternal ACEs had significant and positive total effects on adolescents’ ACEs; (2) Maternal ACEs were indirectly associated with adolescents’ ACEs through maternal parenting stress; (3) Both maternal and paternal ACEs were indirectly associated with adolescents’ ACEs through maternal depressive symptoms and subsequent parenting stress.

### Intergenerational transmission of aces

This study found that both maternal and paternal ACEs were directly associated with adolescents’ ACEs. Consistent with research from Western contexts [6, 39], individuals exposed to childhood maltreatment are at elevated risk of perpetrating maltreatment as caregivers. Parents who have undergone ACEs may learn and apply the ways in which they were treated by their own parents (such as abuse or neglect) to their own parenting styles, thereby increasing the risk of adolescents experiencing ACE themselves. Despite distinctive features of the Chinese sociocultural context, our findings converge with this broader literature, indicating that the intergenerational transmission of ACEs reflects robust processes that generalize across cultural settings.

### Sequential indirect paths of parental depressive symptoms and parenting stress

Firstly, this study found an indirect association between maternal ACEs and adolescents’ ACEs through maternal parenting stress. According to family systems theory, a parent’s unresolved traumas and resultant stress are not merely personal issues but also influencing factors for the entire family system’s dynamics [15]. Mothers with ACEs may struggle with parenting, leading to increased stress levels that can negatively affect the quality of parenting and subsequently raise the likelihood of ACEs in their adolescents. Research demonstrates that maternal depression, often a consequence of unresolved ACEs, is associated with increased parenting stress and less optimal parenting practices [31]. This stress affects parenting quality and, in turn, increases adolescents’ likelihood of experiencing ACEs.

Secondly, maternal ACEs were indirectly associated with adolescents’ ACEs through maternal depressive symptoms and subsequent parenting stress. The parenting stress model [1, 19] underscores the influence of depression on parenting stress, highlighting that parents experiencing depressive symptoms are more likely to face challenges in meeting the demands of parenting. Maternal ACEs can result in emotional regulation difficulties and mental health issues, such as depression [21, 46], which necessitate additional resources to cope with and may result in less emotional support, reduced responsiveness, and inconsistency in parenting behaviors (Kim et al., 2003). Thus, these findings cohesively illustrate that maternal ACEs can engender psychopathological symptoms, amplifying parenting stress and, consequently, leading to ACEs in adolescents.

Thirdly, paternal ACEs were indirectly associated with adolescents’ ACEs through maternal depressive symptoms and subsequent parenting stress. This finding primarily attributed that paternal ACEs influenced maternal depressive symptoms, revealing a “partner effect”. Specifically, maternal ACEs were significantly linked to paternal depressive symptoms, and similarly, paternal ACEs were significantly related to maternal depressive symptoms. In the context of ACEs, research also found that the maternal maltreatment history is associated with paternal affective symptoms [58], which might be because a partner with a history of ACEs may rely more on the other for emotional support and stability. Thus, ACEs may serve as a continuous source of emotional and psychological stress that contributes to the increased likelihood of mutual influence and transmission of depressive symptoms between partners, thereby affecting maternal parenting stress and leading to adolescents’ ACEs.

However, the role of paternal depressive symptoms and parenting stress in the association between paternal ACEs and adolescents’ ACEs was not found. This divergence could be attributed to the distinctive roles that mothers and fathers typically assume in parenting dynamics. Studies indicate that mothers spend significantly more time on parenting than fathers [51, 62], suggesting that the impact of maternal ACEs might be more readily manifested in their parenting behaviors and subsequently affect adolescents’ experiences. Additionally, as the primary caregiver in the family, mothers typically take on more responsibilities for daily care and emotional support [27, 36]. Therefore, their ACEs may have a more direct link to the family emotional dynamics. In addition, mothers traditionally serve as the primary caregivers in China, despite the evolving role of fathers in modern society [43]. Overall, the results highlight the indirect role of fathers in shaping adolescent mental health by influencing maternal depressive symptoms and parenting behaviors. These findings underscore the importance of considering each parent’s unique contributions in understanding the transmission of adverse experiences within families.

## Limitations and implications

The present study is subject to several noteworthy limitations that warrant consideration. First, the self-reporting method used to measure parental ACEs in this study may be susceptible to recall bias. Participants may forget past experiences over time or alter events based on subjective perceptions. This primarily involves underreporting, where individuals who have experienced childhood maltreatment may not report these experiences in adulthood [25, 32]. Therefore, future research could consider employing more objective and diversified measurement methods. Second, the cross-sectional design of this study failed to examine the causality between parental ACEs and adolescent ACEs. Additional longitudinal or other methods such as interviews and observational studies are warranted to further investigate this association. Third, while the current model assumes a unidirectional pathway from parental depressive symptoms to adolescent adversity, parent-child interactions are likely to be bidirectional. Future longitudinal and transactional studies are needed to explore these reciprocal influences. Fourth, participants were recruited from three geographically distinct provinces in China, the present study did not explicitly account for regional variation in the analysis. Future research is needed to examine potential cultural and socioeconomic across regions, as these factors may influence the patterns of intergenerational transmission.

Despite the mentioned limitations in this study, it possesses several strengths. Our study is the large sample situated in the Chinese cultural context, which enhances external validity and allows culturally grounded interpretation of the findings. Theoretically, the research delves into how ACEs are transmitted across generations through parental depressive symptoms and parenting stress, which validates and extends family systems theory and parenting stress model. Practically, these findings offer valuable insights for practitioners. First, clinicians and family therapists can deliver parent education and skills training that cultivate safe, stable family relationships and blunt the effects of childhood adversity [10, 44]. Given that maternal parenting stress is a key conduit of the intergenerational transmission of ACEs, prevention should prioritize stress-reduction components for mothers. In China, online Mindful Parenting provides a feasible template, with demonstrated efficacy [67]. Additionally, the study highlights the need for comprehensive family-based interventions that involve both parents. Supporting fathers in recognizing and addressing the impact of their own ACEs on maternal depressive symptoms, and in turn, the maternal influence on child outcomes, can be a critical strategy in disrupting the intergenerational transmission of ACEs.

## Data Availability

The data supporting this study’s findings are available from the corresponding author upon reasonable request.
